# Supplementary material for: A Comparative Study on the Effect of Euthanasia Methods and Sample Storage Conditions on RNA Yield and Quality in Porcine Tissues
Source: Animals (Basel). 2023 Feb 16;13(4):698. doi: 10.3390/ani13040698 (PMC9952302; doi:10.3390/ani13040698)
Supplement: Supplementary file 1 [file animals-13-00698-s001.zip › animals-2193287-supplementary.pdf]

Supplementary Tables

Table S1. *Number of samples collected from the ten different tissue types, grouped by combining the factors Euthanasia method [Nitrogen Anoxia=>Anox or T-61], Storage Condition [Liquid Nitrogen =>LN$_2$ or RNAlater=> RL] and Time of Sampling [Early or Late]*

	**Euthanasia Method:Storage Condition:Time of Sampling**								
	**Anoxia**				**T61**				
	**RNA*later*™**		**LN$_2$**		**RNA*later*™**		**LN$_2$**		
**Tissue Types**	Anox: RL:Early	Anox: RL:Late	Anox: LN$_2$:Early	Anox: LN$_2$:Late	T-61: RL:Early	T-61: RL:Late	T-61: LN$_2$:Early	T-61: LN$_2$:Late	**Total**
Pituitary*	6	-	-	-	6	-	-	-	**12**
Hypothalamus*	6	-	6	-	6	-	6	-	**24**
Testes	12	12	12	12	12	12	12	12	**96**
Cremaster	12	12	12	12	12	12	12	12	**96**
Deep Ing. Ring*	12	-	12	-	12	-	12	-	**48**
Sup. Ing. Ring*	12	-	12	-	12	-	12	-	**48**
Heart	12	12	12	12	12	12	12	12	**96**
Lungs	12	12	12	12	12	12	12	12	**96**
Liver	12	12	12	12	12	12	12	12	**96**
Kidney	12	12	12	12	12	12	12	12	**96**
**Total**	**108**	**72**	**102**	**72**	**108**	**72**	**102**	**72**	**708**

* Samples were collected only at a single time point (Early), and Pituitary was stored only in RNA*later*™

Table S2. *Overview of the subset of samples for which the RIN values were measured. Samples were grouped by combining the factors Euthanasia method [Nitrogen Anoxia=>Anox or T-61], Storage Condition [Liquid Nitrogen =>LN2 or RNAlater=> RL]*

	**Euthanasia Method:Storage Condition**				
**Tissue Types**	**Anox:RL**	**Anox:LN2**	**T61:RL**	**T61:LN2**	**Total**
Liver	4	4	4	4	**16**
Lungs	4	4	4	3	**15**
Hypothalamus	4	4	4	4	**16**
Pituitary	6	-	6	-	**12**
**Total**	**18**	**12**	**18**	**11**	**59***

*RIN value undetermined for 3 samples and hence only 56 samples with RIN value used in the analysis.

Table S3. *Detailed description of the experimental data grouped by Euthanasia Method*

Characteristic	N	Overall, N = 489[1]	Anoxia, N = 267[1]	T61, N = 222[1]
**Storage**	489			
LN2		244 (50%)	133 (50%)	111 (50%)
RNAlater		245 (50%)	134 (50%)	111 (50%)
**Tissue_Type**	489			
CM		68 (14%)	41 (15%)	27 (12%)
DIR		41 (8.4%)	24 (9.0%)	17 (7.7%)
Heart		55 (11%)	26 (9.7%)	29 (13%)
Hypothalamus		24 (4.9%)	12 (4.5%)	12 (5.4%)
Kidney		76 (16%)	42 (16%)	34 (15%)
Liver		52 (11%)	26 (9.7%)	26 (12%)
Lungs		54 (11%)	28 (10%)	26 (12%)
Pituitary		12 (2.5%)	6 (2.2%)	6 (2.7%)
SIR		36 (7.4%)	20 (7.5%)	16 (7.2%)
Testes		71 (15%)	42 (16%)	29 (13%)
**Time_of_Sampling**	489			
Early		322 (66%)	177 (66%)	145 (65%)
Late		167 (34%)	90 (34%)	77 (35%)
**RNA_Conc**	489	568.64(438.44)	604.56(462.61)	525.45(404.30)
**A260_230 ratio**	489	1.76(0.56)	1.77(0.57)	1.75(0.56)
**A260_280 ratio**	489	2.12(0.04)	2.13(0.04)	2.11(0.04)
**RIN**	56	8.81(1.15)	8.85(1.00)	8.77(1.32)

[1]n (%) – Total number of samples per group and their respective percentages in brackets ;

Mean and SD are denoted for the dependent variables

Table S4. *Detailed description of the experimental data grouped by Storage Condition*

Characteristic	N	Overall, N = 489[1]	LN2, N = 244[1]	RNAlater, N = 245[1]
**Euthanasia_Method**	489			
Anoxia		267 (55%)	133 (55%)	134 (55%)
T61		222 (45%)	111 (45%)	111 (45%)
**Tissue_Type**	489			
CM		68 (14%)	35 (14%)	33 (13%)
DIR		41 (8.4%)	22 (9.0%)	19 (7.8%)
Heart		55 (11%)	27 (11%)	28 (11%)
Hypothalamus		24 (4.9%)	12 (4.9%)	12 (4.9%)
Kidney		76 (16%)	40 (16%)	36 (15%)
Liver		52 (11%)	26 (11%)	26 (11%)
Lungs		54 (11%)	28 (11%)	26 (11%)
Pituitary		12 (2.5%)	0 (0%)	12 (4.9%)
SIR		36 (7.4%)	18 (7.4%)	18 (7.3%)
Testes		71 (15%)	36 (15%)	35 (14%)
**Time_of_Sampling**	489			
Early		322 (66%)	162 (66%)	160 (65%)
Late		167 (34%)	82 (34%)	85 (35%)
**RNA_Conc**	489	568.64(438.44)	525.12(439.57)	611.99(433.88)
**A260_230 ratio**	489	1.76(0.56)	1.66(0.63)	1.86(0.47)
**A260_280 ratio**	489	2.12(0.04)	2.12(0.05)	2.12(0.03)
**RIN**	56	8.81(1.15)	7.99(1.40)	9.26(0.66)

[1]n (%) – Total number of samples per group and their respective percentages in brackets ;
Mean and SD are denoted for the dependent variables

Table S5. *Detailed description of the experimental data grouped by Tissue Type*

Characteristic	N	Overall, N = 489[1]	CM, N = 68[1]	DIR, N = 41[1]	Heart, N = 55[1]	Hypothalamus, N = 24[1]	Kidney, N = 76[1]	Liver, N = 52[1]	Lungs, N = 54[1]	Pituitary, N = 12[1]	SIR, N = 36[1]	Testes, N = 71[1]
**Euthanasia_Method**	489											
Anoxia		267 (100%)	41 (15%)	24 (9.0%)	26 (9.7%)	12 (4.5%)	42 (16%)	26 (9.7%)	28 (10%)	6 (2.2%)	20 (7.5%)	42 (16%)
T61		222 (100%)	27 (12%)	17 (7.7%)	29 (13%)	12 (5.4%)	34 (15%)	26 (12%)	26 (12%)	6 (2.7%)	16 (7.2%)	29 (13%)
**Storage**	489											
LN2		244 (100%)	35 (14%)	22 (9.0%)	27 (11%)	12 (4.9%)	40 (16%)	26 (11%)	28 (11%)	0 (0%)	18 (7.4%)	36 (15%)
RNAlater		245 (100%)	33 (13%)	19 (7.8%)	28 (11%)	12 (4.9%)	36 (15%)	26 (11%)	26 (11%)	12 (4.9%)	18 (7.3%)	35 (14%)
**Time_of_Sampling**	489											
Early		322 (100%)	37 (11%)	41 (13%)	32 (9.9%)	24 (7.5%)	44 (14%)	30 (9.3%)	31 (9.6%)	12 (3.7%)	36 (11%)	35 (11%)
Late		167 (100%)	31 (19%)	0 (0%)	23 (14%)	0 (0%)	32 (19%)	22 (13%)	23 (14%)	0 (0%)	0 (0%)	36 (22%)
**RNA_Conc (ng/µl)**	489	568.64 (438.44)	171.85 (181.56)	195.49 (123.85)	362.67 (177.02)	367.55 (216.26)	938.78 (405.05)	862.42 (353.92)	483.08 (269.81)	896.42 (335.57)	176.04 (116.87)	989.06 (369.35)
**A260_230 ratio**	489	1.75(0.56)	1.29(0.72)	1.55(0.61)	1.66(0.63)	1.82(0.44)	2.01(0.35)	1.87(0.35)	1.85(0.43)	2.16(0.12)	1.54(0.68)	1.98(0.34)
**A260_280 ratio**	489	2.12(0.04)	2.14(0.07)	2.12(0.03)	2.13(0.03)	2.12(0.02)	2.11(0.03)	2.11(0.02)	2.12(0.02)	2.09(0.02)	2.11(0.07)	2.12(0.02)

**RIN (Scale 1-10)**	56	8.81(1.15)	NA(NA)	NA(NA)	NA(NA)	7.56(1.22)	NA(NA)	9.43(0.61)	9.31(0.86)	9.18(0.33)	NA(NA)	NA(NA)

[1]n (%) – Total number of samples per group and their respective percentages are given in brackets ; Mean and SD are denoted for the dependent variables

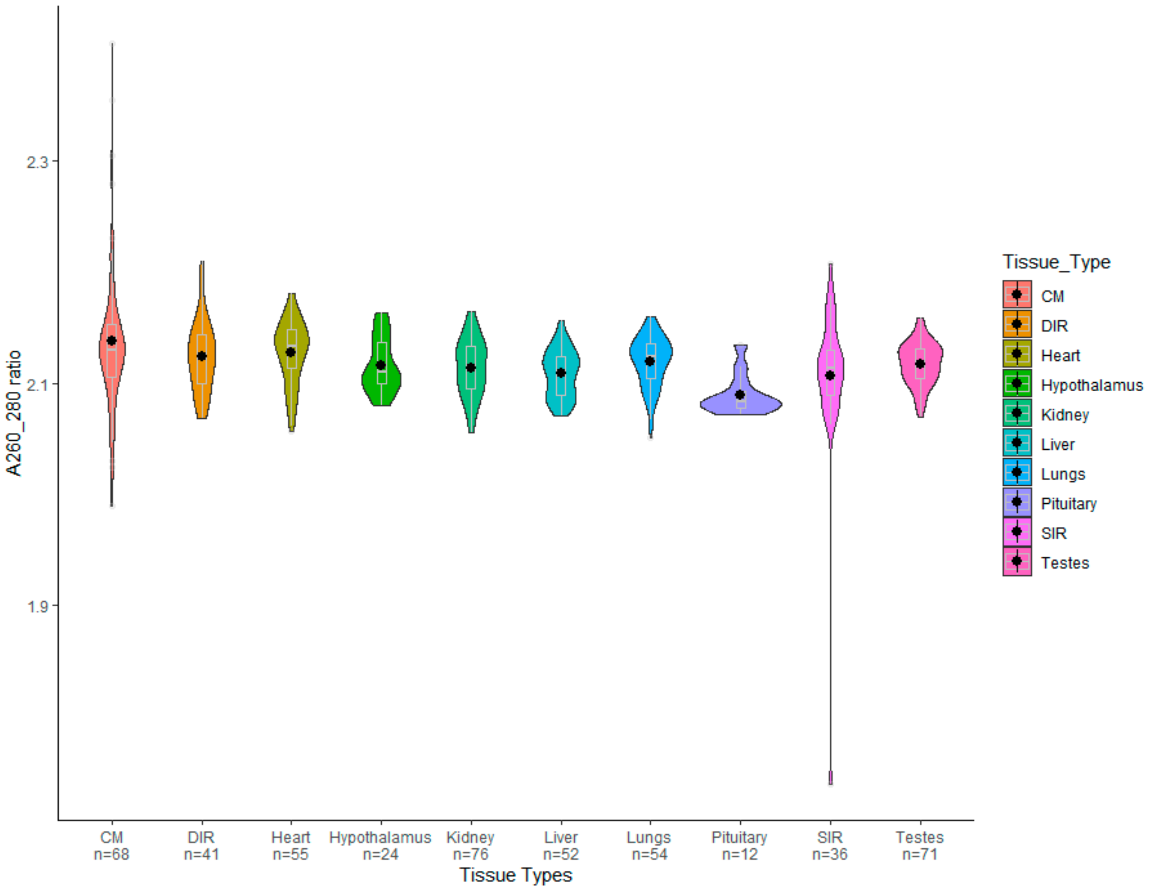

Figure S1. RNA quality based on A260_280 ratio from the ten different tissue types are plotted here. All the tissue types compared

showed good A260_280 ratios, suggestive of pure RNA. CM: Cremaster Muscle; DIR: Deep Inguinal Ring; SIR: Superficial Inguinal Ring

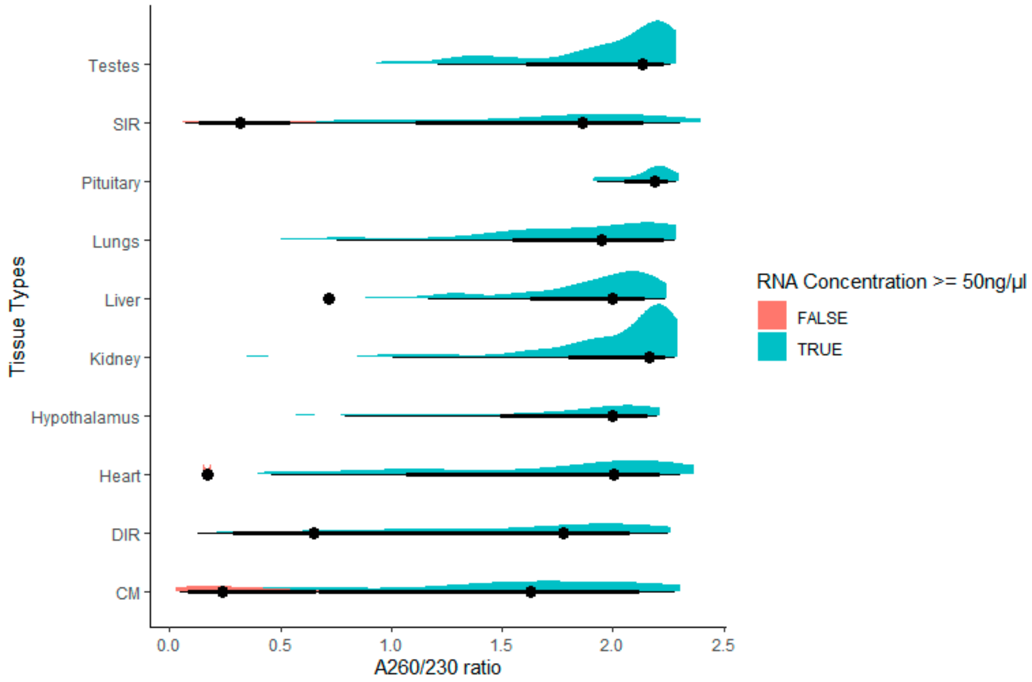

Figure S2. A combined density and interval plot representing RNA concentration and A260/230 ratio in the discarded data and retained data is depicted here. A threshold of 50ng/µl for RNA concentration and 1.0 for A260/230 ratio was set for filtering the data. The mean (black dots) A260/230 ratio for the retained data and discarded data are marked as black dots. CM: Cremaster Muscle; DIR: Deep Inguinal Ring; SIR: Superficial Inguinal Ring

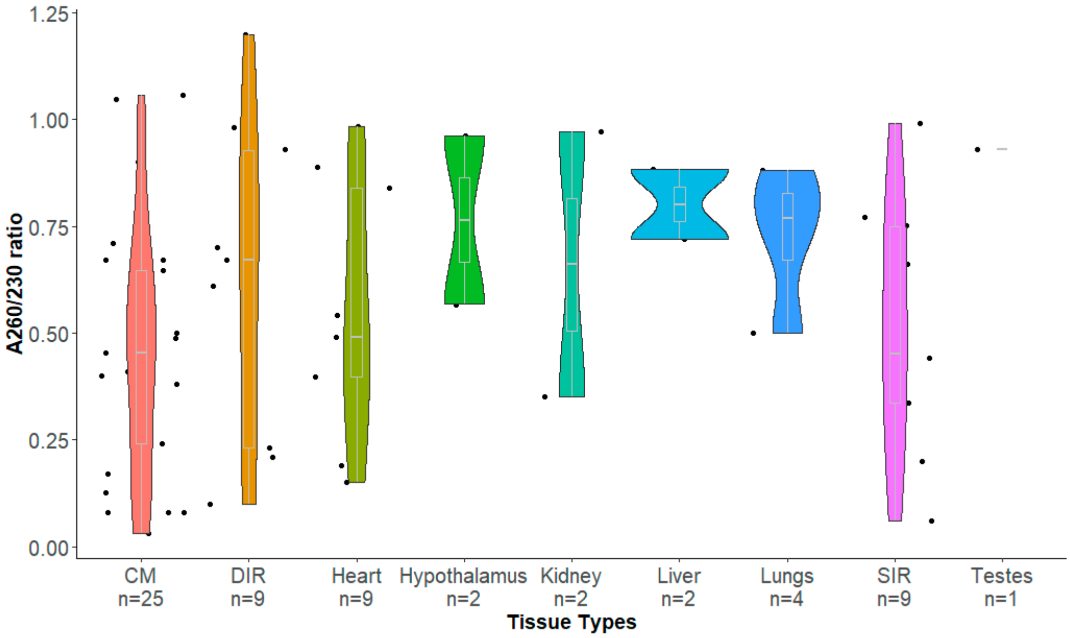

Figure S3. Cremaster muscle sample was the major tissue type discarded due to poor RNA measurements,

while no pituitary samples were discarded. Cremaster muscle (CM), Inguinal rings- Deep (DIR) and Superficial (SIR)

and heart represented the tissue types that had the most samples below the set threshold

Table S6. *Detailed description of the data retained after filtering, grouped by Euthanasia Method*

Characteristic	N	Overall, N = 426[1]	Anoxia, N = 231[1]	T61, N = 195[1]
**Storage**	426			
LN2		197 (46%)	105 (45%)	92 (47%)
RNAlater		229 (54%)	126 (55%)	103 (53%)
**Tissue_Type**	426			
CM		43 (10%)	24 (10%)	19 (9.7%)
DIR		32 (7.5%)	18 (7.8%)	14 (7.2%)
Heart		46 (11%)	20 (8.7%)	26 (13%)
Hypothalamus		22 (5.2%)	12 (5.2%)	10 (5.1%)
Kidney		74 (17%)	41 (18%)	33 (17%)
Liver		50 (12%)	24 (10%)	26 (13%)
Lungs		50 (12%)	28 (12%)	22 (11%)
Pituitary		12 (2.8%)	6 (2.6%)	6 (3.1%)
SIR		27 (6.3%)	17 (7.4%)	10 (5.1%)
Testes		70 (16%)	41 (18%)	29 (15%)
**Time_of_Sampling**	426			
Early		273 (64%)	150 (65%)	123 (63%)
Late		153 (36%)	81 (35%)	72 (37%)
**RNA_Conc**	426	633.29(430.42)	679.60(450.30)	578.43(399.84)
**A260_230 ratio**	426	1.93(0.33)	1.95(0.31)	1.91(0.35)
**A260_280 ratio**	426	2.12(0.03)	2.12(0.03)	2.11(0.02)
**RIN**	55	8.79(1.16)	8.85(1.00)	8.73(1.33)

[1]n (%) – Total number of samples per group and their respective percentages in brackets Mean and SD are denoted for the dependent variables

Table S7. *Detailed description of the data retained after filtering, grouped by Storage Condition*

Characteristic	N	Overall, N = 426[1]	LN2, N = 197[1]	RNAlater, N = 229[1]
**Euthanasia_Method**	426			
Anoxia		231 (54%)	105 (53%)	126 (55%)
T61		195 (46%)	92 (47%)	103 (45%)
**Tissue_Type**	426			
CM		43 (10%)	17 (8.6%)	26 (11%)
DIR		32 (7.5%)	15 (7.6%)	17 (7.4%)
Heart		46 (11%)	21 (11%)	25 (11%)
Hypothalamus		22 (5.2%)	10 (5.1%)	12 (5.2%)
Kidney		74 (17%)	38 (19%)	36 (16%)
Liver		50 (12%)	24 (12%)	26 (11%)
Lungs		50 (12%)	26 (13%)	24 (10%)
Pituitary		12 (2.8%)	0 (0%)	12 (5.2%)
SIR		27 (6.3%)	11 (5.6%)	16 (7.0%)
Testes		70 (16%)	35 (18%)	35 (15%)
**Time_of_Sampling**	426			
Early		273 (64%)	124 (63%)	149 (65%)
Late		153 (36%)	73 (37%)	80 (35%)
**RNA_Conc**	426	633.29(430.42)	618.50(434.95)	646.01(427.02)
**A260_230 ratio**	426	1.93(0.33)	1.91(0.35)	1.95(0.31)
**A260_280 ratio**	426	2.12(0.03)	2.11(0.03)	2.12(0.03)
**RIN**	55	8.79(1.16)	7.99(1.40)	9.25(0.66)

[1]n (%) – Total number of samples per group and their respective percentages in brackets

Mean and SD are denoted for the dependent variables

Table S8. *Detailed description of the data retained after filtering, grouped by Tissue Type*

Characteristic	N	Overall, N = 426[1]	CM, N = 43[1]	DIR, N = 32[1]	Heart, N = 46[1]	Hypothalamus, N = 22[1]	Kidney, N = 74[1]	Liver, N = 50[1]	Lungs, N = 50[1]	Pituitary, N = 12[1]	SIR, N = 27[1]	Testes, N = 70[1]
**Euthanasia_Method**	426											
Anoxia		231 (100%)	24 (10%)	18 (7.8%)	20 (8.7%)	12 (5.2%)	41 (18%)	24 (10%)	28 (12%)	6 (2.6%)	17 (7.4%)	41 (18%)
T61		195 (100%)	19 (9.7%)	14 (7.2%)	26 (13%)	10 (5.1%)	33 (17%)	26 (13%)	22 (11%)	6 (3.1%)	10 (5.1%)	29 (15%)
**Storage**	426											
LN2		197 (100%)	17 (8.6%)	15 (7.6%)	21 (11%)	10 (5.1%)	38 (19%)	24 (12%)	26 (13%)	0 (0%)	11 (5.6%)	35 (18%)
RNAlater		229 (100%)	26 (11%)	17 (7.4%)	25 (11%)	12 (5.2%)	36 (16%)	26 (11%)	24 (10%)	12 (5.2%)	16 (7.0%)	35 (15%)
**Time_of_Sampling**	426											
Early		273 (100%)	21 (7.7%)	32 (12%)	26 (9.5%)	22 (8.1%)	43 (16%)	29 (11%)	27 (9.9%)	12 (4.4%)	27 (9.9%)	34 (12%)
Late		153 (100%)	22 (14%)	0 (0%)	20 (13%)	0 (0%)	31 (20%)	21 (14%)	23 (15%)	0 (0%)	0 (0%)	36 (24%)
**RNA_Conc**	426	633.29 (430.42)	238.67 (197.29)	222.37 (123.78)	392.78 (164.17)	372.47 (223.78)	957.29 (394.06)	885.46 (336.55)	498.30 (269.91)	896.42 (335.57)	208.94 (113.81)	995.91 (367.45)

**A260_230 ratio**	426	1.93 (0.33)	1.77 (0.35)	1.81 (0.36)	1.88 (0.41)	1.92 (0.31)	2.04 (0.27)	1.92 (0.28)	1.94 (0.30)	2.16 (0.12)	1.88 (0.33)	1.99 (0.31)
**A260_280 ratio**	426	2.12 (0.03)	2.12 (0.04)	2.12 (0.03)	2.12 (0.03)	2.11 (0.02)	2.11 (0.03)	2.11 (0.02)	2.12 (0.02)	2.09 (0.02)	2.11 (0.03)	2.12 (0.02)
**RIN**	55	8.79 (1.16)	NA(NA)	NA(NA)	NA(NA)	7.56 (1.22)	NA(NA)	9.43 (0.61)	9.27 (0.88)	9.18 (0.33)	NA(NA)	NA(NA)

[1]n (%) – Total number of samples per group and their respective percentages in brackets ; Mean and SD are denoted for the dependent variables

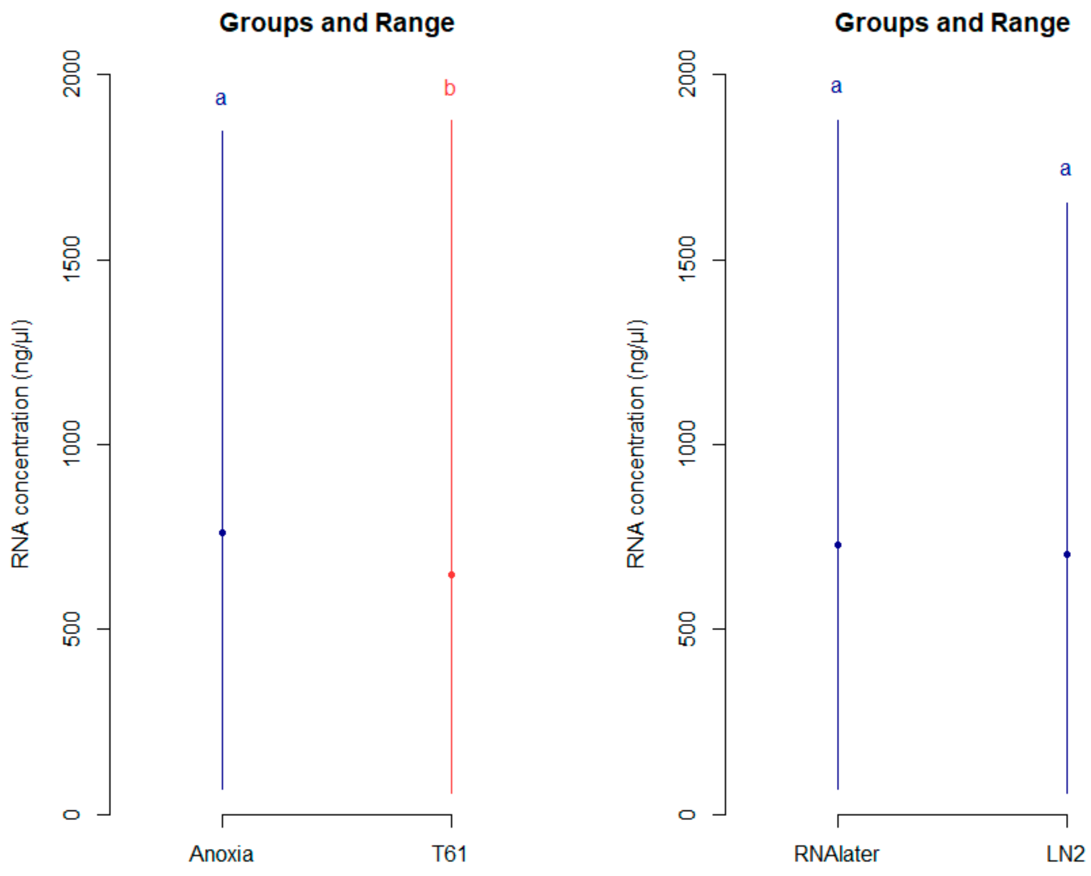

Figure S4. Comparison of mean RNA concentrations between Euthanasia Methods and Storage conditions. Results from the post hoc analysis confirms the significant differences between the Anoxia and T-61 euthanasia methods. Range of RNA concentration (bars) and means (dots) per euthanasia method and storage condition are marked in the figure. Groups carrying different superscript letters and colours differ significantly ($p<0.05$)

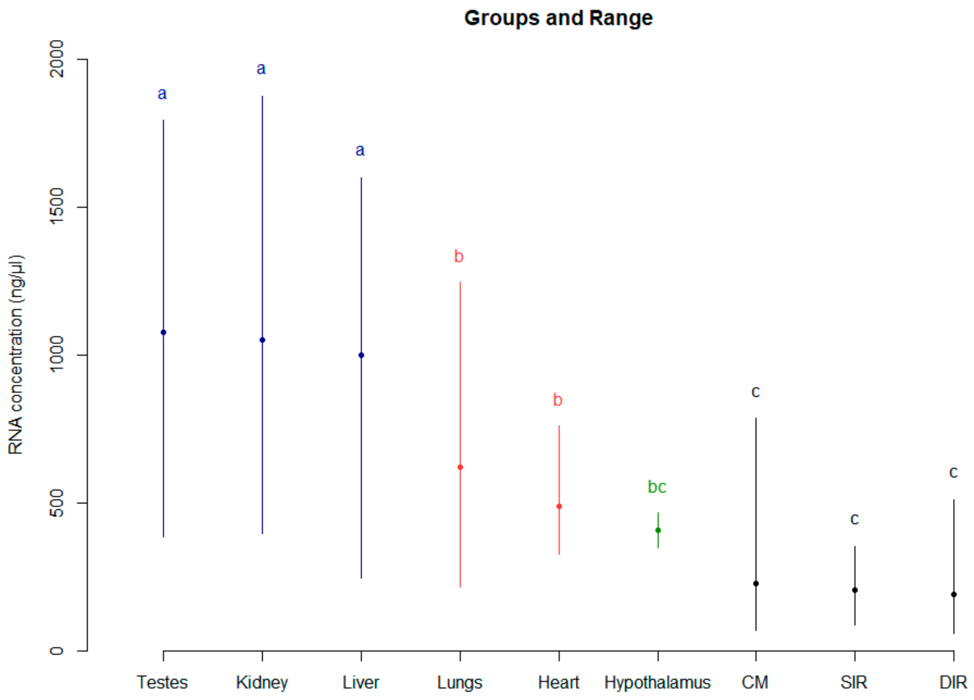

Figure S5. Results from the post hoc analysis is plotted and it confirms the significant differences between various tissue types, based on comparison of the respective average RNA concentrations. Range of RNA concentration (bars) and means (dots) per tissue types are marked in the figure. Groups carrying different superscript letters and colours differ significantly ($p<0.05$)

Histogram of residuals on the model for A260/230 ratio

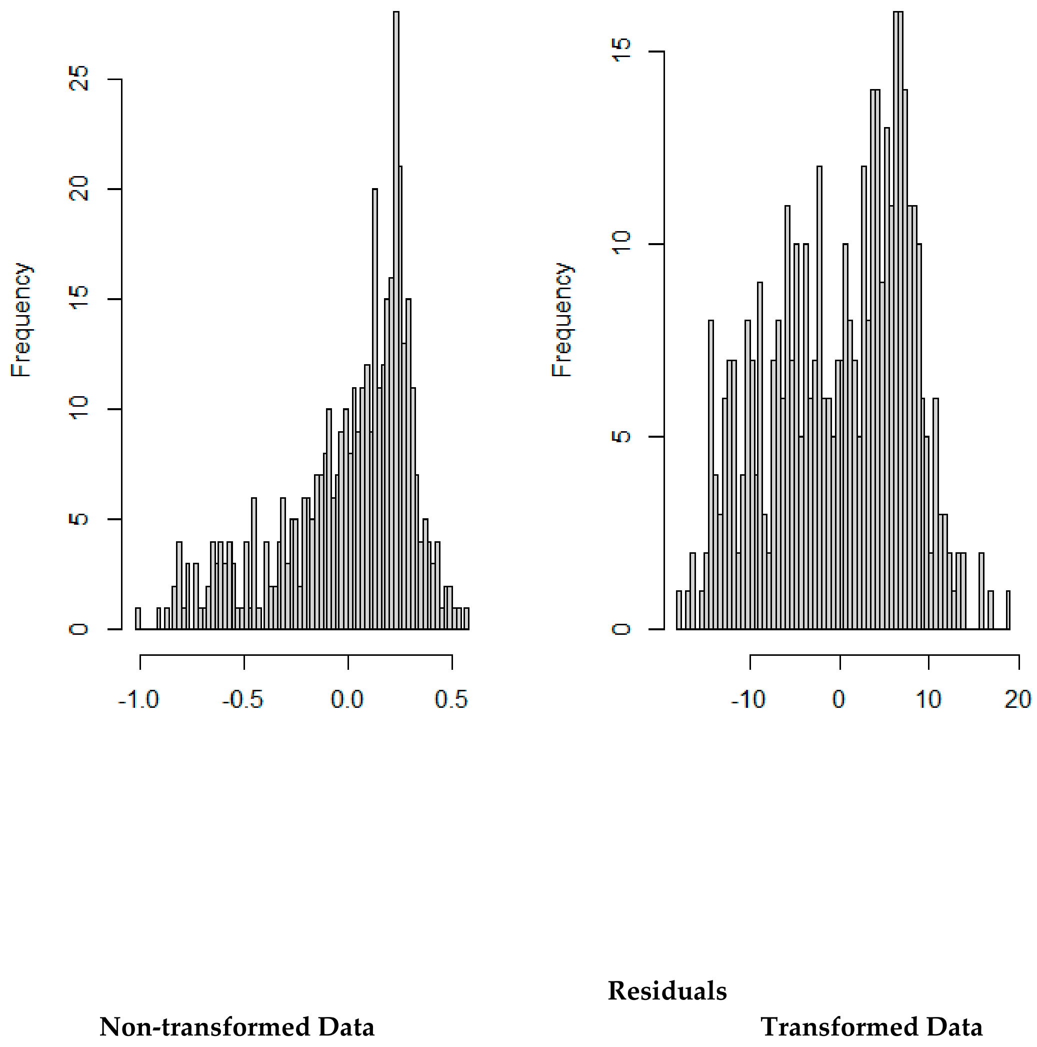

Figure S6. Effect of transformation on the residuals. Histogram of residuals on the regression model with A260/230 ratio (Non transformed vs Transformed)

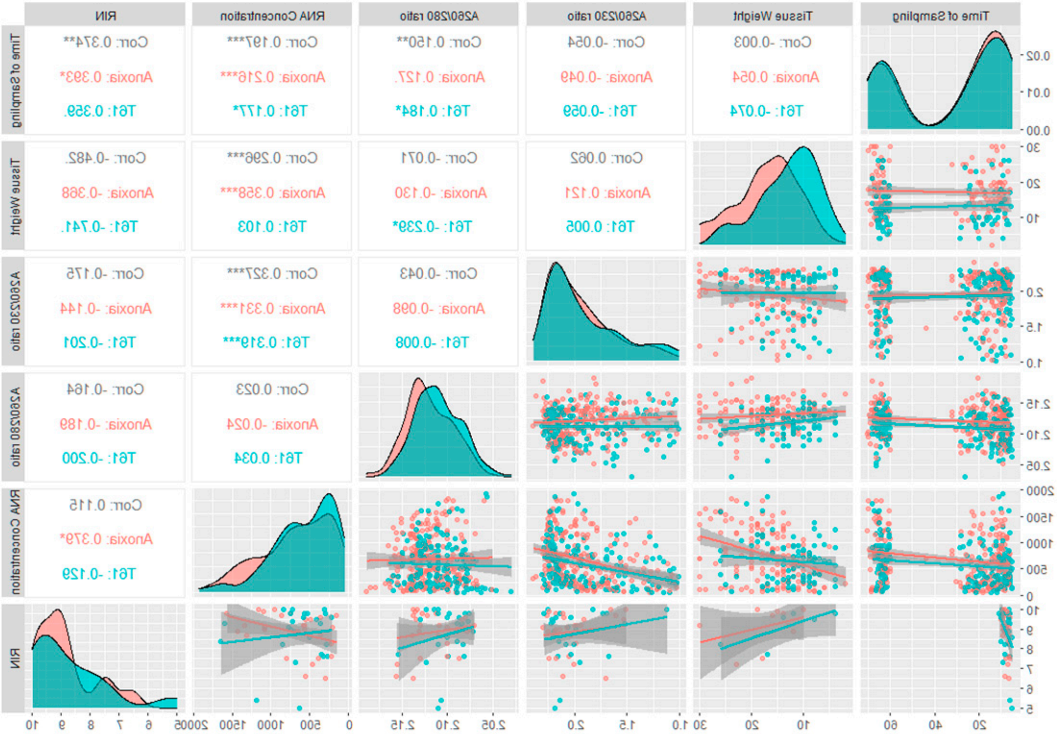

Figure S7. Pairs plot constructed using euthanasia method as the grouping variable.

Euthanasia method had no significant effect on A260/230 ratio

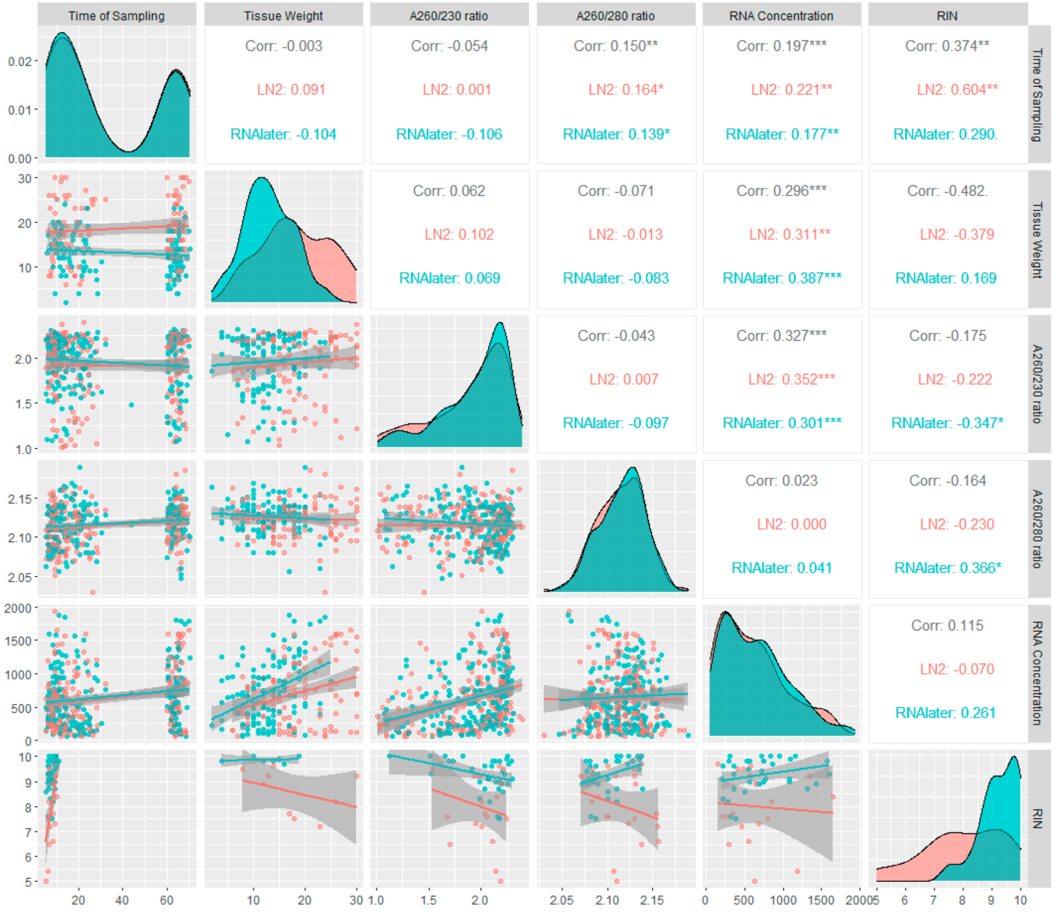

Figure S8. Pairs plot constructed using storage condition as the grouping variable. Storage condition had no significant effect on A260/230 ratio

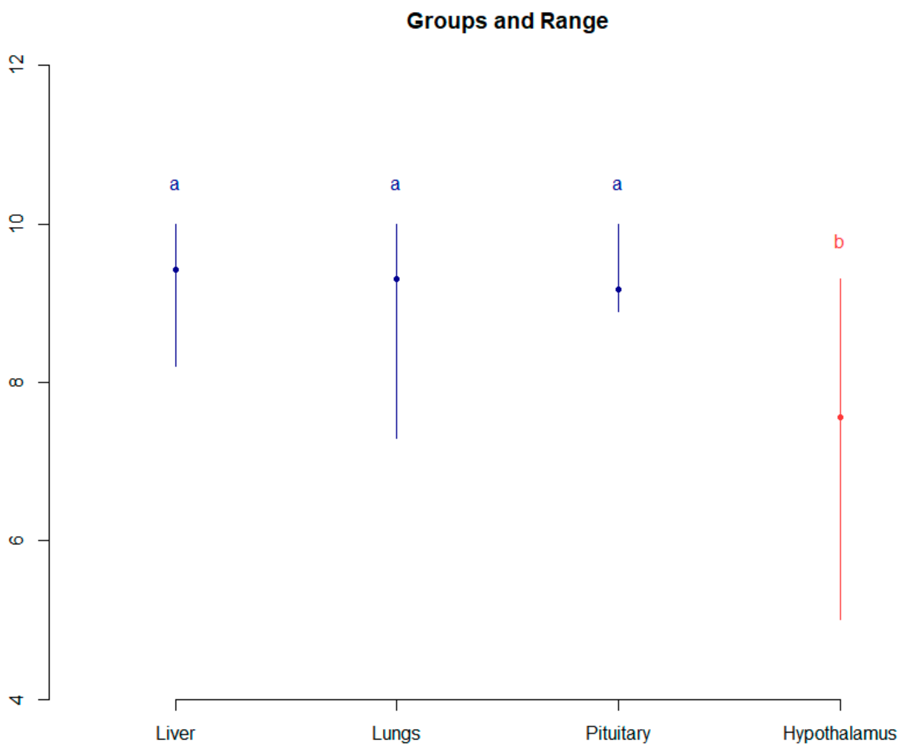

Figure S9. Results from the post hoc analysis is plotted here and it confirms the significant differences between various tissue types, based on comparison of the respective average RIN values. Range of RIN values and means (dots) per tissue types are marked in the figure. Groups carrying different superscript letters and colours differ significantly ($p<0.05$)
